# Increased Direct Current-Electroencephalography Shifts During Induction of Anesthesia in Elderly Patients Developing Postoperative Delirium

**DOI:** 10.3389/fnagi.2022.921139

**Published:** 2022-06-28

**Authors:** Victoria Windmann, Jens P. Dreier, Sebastian Major, Claudia Spies, Gunnar Lachmann, Susanne Koch

**Affiliations:** ^1^Department of Anesthesiology and Operative Intensive Care Medicine (CCM, CVK), Charité – Universitätsmedizin Berlin, Corporate Member of Freie Universität Berlin, Humboldt-Universität zu Berlin, Berlin, Germany; ^2^Center for Stroke Research Berlin, Charité – Universitätsmedizin Berlin, Corporate Member of Freie Universität Berlin, Humboldt-Universität zu Berlin, Berlin, Germany; ^3^Department of Experimental Neurology, Charité – Universitätsmedizin Berlin, Corporate Member of Freie Universität Berlin, Humboldt-Universität zu Berlin, Berlin, Germany; ^4^Department of Neurology, Charité – Universitätsmedizin Berlin, Corporate Member of Freie Universität Berlin, Humboldt-Universität zu Berlin, Berlin, Germany; ^5^Bernstein Center for Computational Neuroscience Berlin, Berlin, Germany; ^6^Einstein Center for Neurosciences Berlin, Berlin, Germany

**Keywords:** DC-EEG, blood–brain barrier, postoperative delirium (POD), hyperventilation, anesthesia induction, direct current EEG, electroencephalography

## Abstract

**Background:**

Changes in the direct current (DC) electroencephalography (EEG), so-called DC shifts, are observed during hypoxia, hypo-/hypercapnia, anesthetic administration, epileptic seizures, and spreading depolarizations. They are associated with altered cerebral ion currents across cell membranes and/or the blood–brain barrier (BBB). Here, we measured DC shifts in clinical practice during hyperventilation (HV) and anesthesia induction, and investigated whether such DC shifts correlate with the occurrence of postoperative delirium (POD) in older patients.

**Methods:**

In this prospective observational study (subproject of the BioCog study, NCT02265263; EA2/092/14), a continuous pre- and perioperative DC-EEG was recorded in patients aged ≥65 years. The preoperative DC-EEG included a 2 min HV with simultaneous measurement of end-tidal CO_2_. Of the perioperative recordings, DC-EEG segments were chosen from a 30 s period at the start of induction of anesthesia (IOA), loss of consciousness (LOC), and during a stable anesthetic phase 30 min after skin incision (intraOP). The DC shift at Cz was determined in μV/s. All patients were screened twice daily for the first seven postoperative days for the occurrence of POD. DC-EEG shifts were compared in patients with (POD) and without postoperative delirium (noPOD).

**Results:**

Fifteen patients were included in this subproject of the BioCog study. DC shifts correlated significantly with concurrent HV, with DC shifts increasing the more end-tidal CO_2_ decreased (*P* = 0.001, Spearman’s rho 0.862). During the perioperative DC-EEG, the largest DC shift was observed at LOC during IOA. POD patients (*n* = 8) presented with significantly larger DC shifts at LOC [POD 31.6 (22.7; 38.9) μV/s vs. noPOD 4.7 (2.2; 12.5) μV/s, *P* = 0.026].

**Conclusion:**

DC shifts can be observed during HV and IOA in routine clinical practice. At anesthesia induction, the DC shift was greatest at the time of LOC, with POD patients presenting with significantly stronger DC shifts. This could indicate larger changes in gas tensions, hypotension and impaired cerebral autoregulation or BBB dysfunction in these patients.

**Clinical Trial Registration:**

www.clinicaltrials.gov, identifier NCT02265263.

## Introduction

Direct current (DC)-electroencephalography (EEG) shifts can be observed during various pathophysiological states such as cerebral hypoxia, spreading depolarization (SD), and epileptic seizures, as well as during alterations to the cerebral blood flow (CBF), hypoxia, and hyper- or hypocapnia (Lehmenkuhler et al., 1999; Vanhatalo et al., 2003; Voipio et al., 2003; Drenckhahn et al., 2012). Moreover, a study in cats revealed DC shifts in response to the administration of thiopental/isoflurane (Nita et al., 2004).

Up to today the origins of these DC shifts are not fully understood and subject to ongoing scientific debate.

Direct current shifts mediated by hypo-/hypercapnia or hypoxia in rats are thought to be of non-neuronal origin generated form an altered electrical polarization across the blood–brain barrier (BBB) representing a diffusion potential determined by the proton gradient between the intravascular and extracellular compartment (Tschirgi and Taylor, 1958; Voipio et al., 2003; Kang et al., 2013). It has to be noted that there are species differences in the trans-BBB potential (Besson et al., 1970; Woody et al., 1970). Cats and monkeys show fundamentally different CO_2_-dependent intracortical DC shifts compared to rats, rabbits, goats, and dogs. These are nevertheless assumed to be generated at the BBB but appear to be caused by changes in CBF rather than by the proton gradient across the barrier (Besson et al., 1970; Woody et al., 1970; Nita et al., 2004).

Although the electrical barrier of the BBB is very robust, it has been suggested that DC shifts could be used to monitor the integrity of the BBB (Kiviniemi et al., 2017). A dysfunction of the BBB may be involved in the pathophysiology of postoperative delirium (POD), one of the most frequent neurocognitive complications after surgery. Hughes et al. (2016) found that plasma markers for endothelial activation and BBB injury, such as plasminogen activator inhibitor-1, E-selectin, and S100 were independently associated with the duration of delirium.

Scientific findings on DC shifts are largely based on *in vitro* data and from animal experiments or experimental data in healthy subjects. Therefore, the aim of this study was to detect DC shifts in patients in routine clinical practice. We first recorded DC-EEGs under the influence of hyperventilation (HV) to prove the feasibility of our set-up. Second, we determined the occurrence of DC shifts during anesthesia induction and third, we investigated a possible correlation between these DC shifts and the development of POD.

## Materials and Methods

### Study Participants and Groups

This proof of concept study was performed as a subproject of the BioCog study between July 2016 and December 2016 at the study site Charité – Universitätsmedizin Berlin Campus Virchow-Klinikum, Germany (NCT02265263). Ethics approval was obtained from the institutional review board (EA2/092/14) and written informed consent was obtained from all patients. This clinical trial meets the requirements set out by the ICH-GCP and Declaration of Helsinki.

Patients were eligible for the study if they were aged 65 years or older and planned to undergo elective surgery under general anesthesia with an expected operative time of at least 60 min. Exclusion criteria comprised a preoperative Mini-Mental-State-Examination below 24 points, neuropsychiatric morbidity, which limits the conduction of neurocognitive testing, anacusis or hypoacusis, proposed neurological surgery, and neurological preconditions, i.e., a history of seizures or stroke. Moreover, patients with a history of pulmonary diseases associated with hypercapnia, patients who received a narcotic other than propofol (i.e., thiopental) for induction of anesthesia (IOA) and patients whose EEG showed too many artifacts were excluded from further analyses.

Baseline demographic data including patient history, other comorbidities, and long-term medication, were obtained on the day of inclusion by reviewing the medical records. The chart review was done by clearly stated criteria: the study personal screened the patients’ charts for any situation mentioned by nurses or doctors in charge indicating that the patient was “confused, agitated, drowsy, delirious, or received any antipsychotic medication.” Since POD has a fluctuating course, the chart review was performed not to miss any delirious situations of each patient.

In accordance with the observational character of this trial, medication for induction and maintenance of anesthesia was not part of the study protocol and chosen according to clinical needs as determined by the anesthetist in charge. All patients received guideline-based anesthesiological and surgical treatment according to our standard operating procedures (SOPs) (Spies et al., 2013).

Some of the patients received oral premedication with midazolam 30 min prior to IOA to reduce anxiety or stress. Whether patients received midazolam was determined by the anesthesiologist who conducted the anesthesiology educational interview the day before surgery.

The surgeries performed during this trial comprised predominantly abdominal surgeries (*n* = 11) and some musculoskeletal and vascular surgeries (*n* = 4). A detailed summary of the performed surgeries is given in Supplementary Table 1.

All patients included in this subproject were planned to receive an arterial catheter for clinical reasons such as anticipated high blood loss or need for continuous monitoring of blood pressure. They received repetitive arterial blood gas analyses immediately after an arterial line was established until the end of surgery. We determined the first arterial pCO_2_ after IOA.

C-reactive protein (CRP) values obtained preoperatively on the day of surgery were compared to investigate a relationship between the inflammatory state and DC shifts.

### Electroencephalography Recordings

Direct current-electroencephalography were recorded using a DC-EEG amplifier and DC-stable Ag/AgCl electrodes placed on the patients’ head in accordance with the 10/20 system at Fp1, Fp2, T3, T4, Cz, O1, and O2 with reference electrode at Fpz and the earth electrode on the ear lobe (BrainVision Recorder, Brain Products GmbH). Supplementary Figure 1 illustrates the electrode placement. In order to reduce skin impedance and artifacts caused by galvanic skin responses the patients’ skin beneath the electrodes was prepped with alcohol and scratched with abrasive and conductive skin preparation gel. Impedance was kept below 5 kΩ.

On the day before surgery a preoperative DC-EEG including a 2-min HV period was recorded. Following a 2-min baseline or resting-state recording during which the patients were asked to keep their eyes closed and breathe normally, patients were asked to hyperventilate for 2 min by increasing the rate and depth of breathing. During the baseline and HV phase end-tidal CO_2_ (etCO_2_) was determined *via* a canopy hood of an indirect calorimeter usually used to estimate the energy expenditure of ICU patients (Ashcraft and Frankenfield, 2015).

Recordings of the perioperative DC-EEGs started prior to IOA at least 30 s before administration of the first drug and lasted throughout the entire surgery. Markers were placed in the DC-EEG files to indicate a DC-EEG period before IOA (baseline), administration of the first drug for IOA, loss of consciousness (LOC) marked by the loss of eye-lid reflex (LOC), and a stable phase during anesthesia 30 min after the beginning of surgery (intraOP). A total of 30 s time windows were chosen for further analyses. During IOA the loss of eye-lid reflex was tested every 5 s after patients had closed their eyes.

### Direct Current-Electroencephalography Analysis

As the DC-EEG is susceptible to external influences such as skin resistance, the respective baseline DC-EEG of each individual patient was used to subtract these influences. As a result, the DC-EEG was “calibrated” and mainly the dynamics in the respective segments were examined. We determined the absolute value of the amplitude of DC shifts (in μV) as well as the absolute value of the slope of the DC shifts (in μV/s) in LabChart (ADInstruments Ltd.) and subtracted the previously measured “baseline drift,” i.e., the amplitude or slope of the respective baseline DC-EEG segment.

We then further analyzed the “calibrated” DC shifts

1. During a 2-min segment during HV
2. During a 30 s segment after IOA
3. During a 30 s segment around LOC
4. During a 30 s segment intraOP.

**FIGURE 1:**
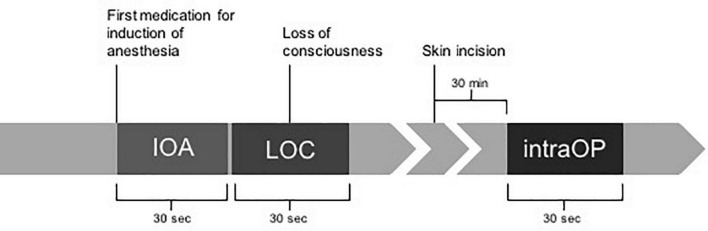
Timeline of chosen EEG segments during perioperative EEGs. A total of 30-second EEG segments were selected for further analyses based on different marked time points during anesthesia induction and surgery. Markers comprised administration of the first drug for induction of anesthesia (IOA), loss of consciousness marked by the loss of eye-lid reflex (LOC), and a stable phase during anesthesia 30 min after the beginning of surgery marked by incision of the skin (intraOP).

Figure 1 shows how the EEG segments were chosen.

The time constants during induction were not uniform among patients. In some cases, rapid sequence inductions were performed so that the time between IOA and LOC was shorter than 30 s. Therefore, to still be able to compare patients, we normalized the amplitude over time and referred to it as “DC shifts.” In all our analyses, we focused on the DC shifts measured at the Cz electrode, as previous studies suggested that the largest DC shift occurred here (Voipio et al., 2003).

### Postoperative Delirium

Patients were assessed for the occurrence of POD twice daily from postoperative day 1 through 7 by trained study staff. POD was defined according to the 5th edition of the Diagnostic and Statistical Manual of Mental Disorders (DSM-5) criteria. Patients were considered delirious in case of: ≥2 cumulative points on the nursing Delirium Screening Scale (Nu-DESC) and/or a positive Confusion Assessment Method (CAM) score and/or a positive CAM for the Intensive Care Unit (CAM-ICU) score and/or patient chart review that shows descriptions of delirium (e.g., confused, agitated, drowsy, disoriented, delirious, received antipsychotic therapy).

### Blood Analyses

Patients’ blood was drawn on the day of surgery prior to IOA. We determined inflammatory parameters [CRP and interleukin-6 (IL-6)], as well as markers associated with the integrity of the BBB (S-100, endocan, zonulin) and correlated them with the amplitude of the DC shifts observed during IOA.

### Outcome Parameters

The primary outcome of this study was to determine whether DC shifts occurred in response to HV and IOA in humans during routine clinical practice. A secondary outcome parameter was the magnitude of DC shifts in patients with and without POD.

### Statistical Analysis

As this is a subproject of a bigger trial, patients were included according to the sample size calculation for the BioCog study. There have been no previous trials investigating the underlying statistical hypotheses (association between DC shifts and POD), the subsequent statistical tests are therefore only to be understood as exploratory ones.

Numerical calculations were performed with IBM© SPSS© Statistics, version 27. Data are expressed as median (25%, 75% quartiles), or *n* frequencies (%). After exploratory data analysis, all tests were performed by non-parametric Mann–Whitney *U* tests for group comparisons of continuous variables and exact Chi-squared-test for qualitative data. A one-way repeated measures ANOVA was used to compare the DC shifts in different EEG-segments during IOA. Moreover, Spearman’s correlation was performed to compare continuous variables. A two-tailed *P*-value < 0.05 was considered statistically significant.

## Results

### Study Population

**FIGURE 2:**
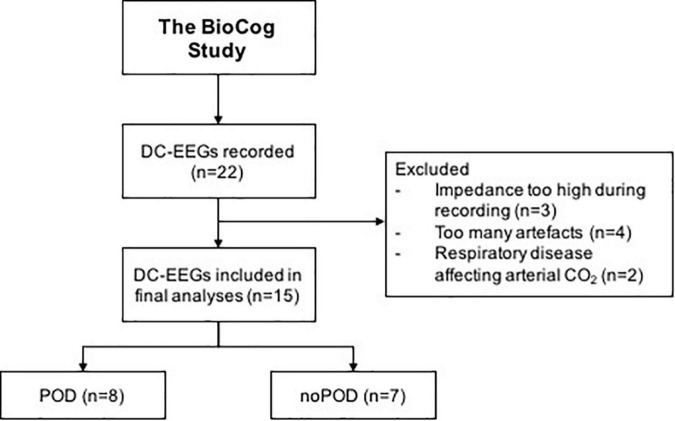
Consort diagram.

A total of 22 patients was included in this subtrial of the BioCog study, however, only 15 patients met the final inclusion criteria and were included in the final analyses. A consort diagram is shown in Figure 2. Baseline characteristics of all patients are included in Table 1.

**TABLE 1:** Patient characteristics for the full sample, POD, and noPOD patients.

	Full sample (*n* = 15)	POD (*n* = 8)	noPOD (*n* = 7)	*P*-value
Age (years)	72 (70; 76)	73 (71; 76)	70 (65; 76)	0.281*
Female *n* (%)	7 (46.7)	4 (50.0)	3 (42.9)	0.782^†^
BMI (kg.m^–2^)	25.0 (22.3; 27.9)	24.6 (22.0; 26.7)	26.6 (24.3; 28.0)	0.336*
Midazolam premedication *n* (%)	4 (26.7)	2 (25.0)	2 (28.6)	0.876^†^
ASA-score				0.185^†^
II *n* (%)	4 (26.7)	1 (12.5)	3 (42.9)	
III *n* (%)	11 (73.3)	7 (87.5)	4 (57.1)	
Preoperative CCI	2 (1.0; 3.0)	2 (0.5; 2.8)	2 (1.0; 3.0)	1.000*
Preoperative CRP (mg/L)	8.4 (1.5; 34.6)	7.1 (1.3; 32.8)	8.4 (2.3; 99.5)	0.792*
**Intraoperative parameters**				
Duration of surgery (min)	248 (131; 353)	323 (187; 413)	133 (118; 303)	0.232*
Propofol dosage at induction (mg)	150 (150;180)	150 (150; 188)	150 (150; 180)	0.779*
Total intravenous/Volatile anesthesia *n* (%)	2 (13.3)/13 (86.7)	1 (12.5)/7 (87.5)	1 (14.3)/6 (85.7)	0.919^†^
Fentanyl dosage at induction (μg)	200 (220; 213) (*n* = 14)	200 (200; 250) (*n* = 7)	200 (200; 200) (*n* = 14)	0.805*
Remifentanil dosage at induction (μg/kg/min)	0.2 (*n* = 1)	0.2 (*n* = 1)	–	–
Esketamine during intraOP segment *n* (%)	3 (20)	2 (25)	1 (14)	0.605^†^
First intraoperative paCO_2_ (mmHg)	39.8 (34.8; 44.4)	39.8 (37.5; 45.7)	36.6 (27.4; 43.9)	0.295*
**Outcome parameters**				
Hospital length of stay^+^ (days)	9.5 (6.5; 14.3)	12.0 (9.0; 22.0)	8 (5.5; 10.5)	0.106*
ICU length of stay (days)	1.0 (1.0; 4.0)	1.0 (1.0; 15.0)	1.0 (0.8; 2.8)	0.628*

Data are shown as median with quartiles (25%; 75%) or as frequencies n (%). P-values were calculated using the Chi-square test^†^ or Wilcoxon–Mann–Whitney U test*, respectively.

*ASA, American Society of Anesthesiologists; BMI, body mass index; CCI, Charlson’s Comorbidity Index; CRP, C-reactive protein; ICU, Intensive Care Unit.*

*^+^Without deaths.*

Anesthesia was induced with propofol in all patients. Analgesia was performed with either fentanyl or remifentanil. Maintenance of anesthesia was performed with propofol or volatile anesthetics, either sevoflurane or desflurane.

### Electroencephalography During Hyperventilation

**FIGURE 3:**
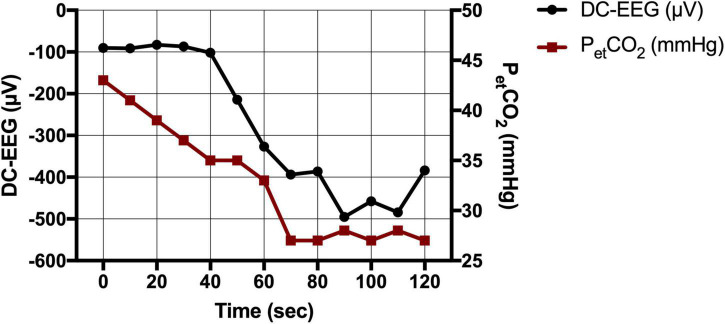
Direct current-electroencephalography and end-tidal CO_2_ during hyperventilation (HV). CO_2_ drop as measured *via* the end-tidal CO_2_ (p_et_CO_2_ in mmHg) and simultaneous DC shift (in μV) during HV in an exemplary patient.

**FIGURE 4:**
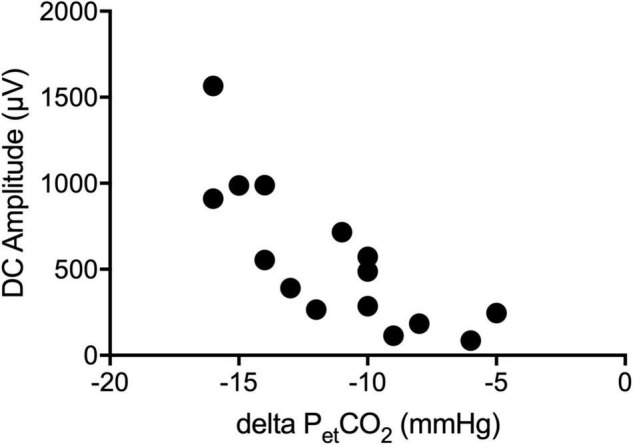
Correlation of the DC-EEG and end-tidal CO_2_. The amplitude of the DC shift during hyperventilation (HV) increased the more the end-tidal CO_2_ (p_et_CO_2_) decreased.

Fifteen patients received a preoperative DC-EEG recording with simultaneous HV. The end-tidal CO_2_ decreased by 11 (Held et al., 1964; Woody et al., 1970) mmHg on average. All patients showed a shift of the DC-EEG at Cz during the HV phase. While most patients showed negative DC deflections (*n* = 12), some also presented with a positive shift (*n* = 3). The median DC amplitude across all patients without regard of the polarity was 487.0 (183.7; 910.7) μV. The amplitude of this DC shift increased the more the end-tidal CO_2_ (p_et_CO_2_) decreased (Spearman’s rho 0.862, *P* = 0.001) (Figures 3, 4). The median DC shift per decrease of 1 mmHg p_et_CO_2_ was 39.5 (23.0; 65.1) μV/mmHg. When comparing HV-induced DC shifts in patients with and without POD, no significant differences could be observed. Even though the median DC shift in POD was larger, there was no significant difference during 2 min of HV [DC amplitude noPOD 285.0 (148.6; 634.7) μV vs. POD 741.5 (386.7; 1132.2) μV, *P* = 0.224].

### Perioperative Electroencephalography

**FIGURE 5:**
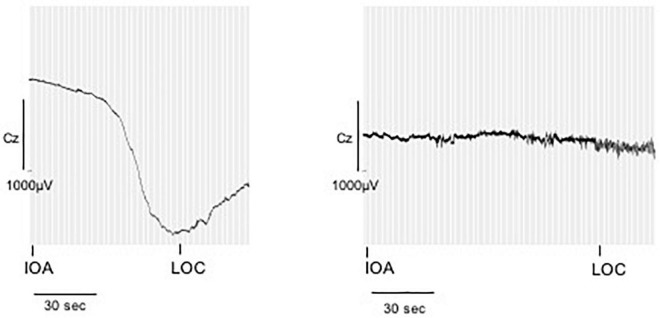
Direct current shifts during anesthesia induction. DC shifts of two example patients during anesthetic induction. The marker IOA represents the onset of narcotic administration, LOC represents loss of consciousness, here defined as loss of the eyelid closure reflex. While some patients presented with marked DC shifts in response to IOA, especially around LOC, others showed only small drifts of the DC potential.

**FIGURE 6:**
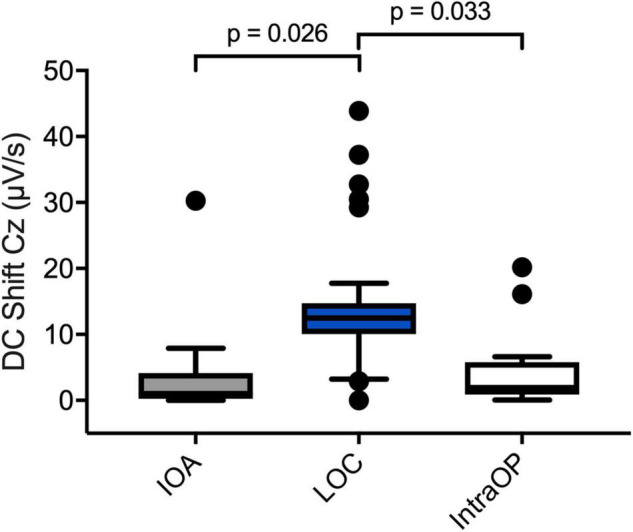
Perioperative DC shifts at different time points. Boxplots depicting DC shifts averaged over all patients during IOA (*n* = 13), LOC (*n* = 12), and intraOP (*n* = 13). The shift at the LOC segment was significantly larger than during the IOA or intraOP segment. A one-way repeated measures analysis of variance test was used to compare the DC shifts.

Fifteen perioperative EEGs were included for further analyses. Similar to the preoperative recordings during HV patients displayed DC shifts with both positive and negative polarity in response to IOA, at LOC and during the intraOP EEG segment (Figure 5). While some patients presented with marked DC shifts in response to IOA, especially around LOC, others showed only small drifts of the DC potential. Three patients showed large negative DC deflections that changed polarity and returned toward baseline (Figure 5). At IOA the median slope without regard of the polarity of the DC shift at Cz was 1.0 (0.2; 4.2) μV/s. At LOC significantly larger DC shifts, in median 13.1 (3.0; 32.2) μV/s, could be observed as compared to IOA (*P* = 0.026). During the intraOP time epoch, the DC shift decreased to a median of 1.9 (0.9; 5.8) μV/s again, thus, being significantly smaller as compared to LOC (*P* = 0.033) (Table 2 and Figure 6).

**TABLE 2:** Direct current shifts at different time points during induction of anesthesia.

	Full sample	POD	noPOD	*P*-value
DC shift IOA (μV/s)	1.0 (0.2; 4.2)	2.5 (1.0; 5.4)	0.4 (0.0; 2.7)	0.138
DC shift LOC (μV/s)	13.1 (3.0; 32.2)	31.6 (22.7; 38.9)	4.7 (2.2; 12.5)	0.026*
DC shift intraOP (μV/s)	1.9 (0.9; 5.8)	1.9 (0.2; 5.0)	3.1 (1.0; 9.0)	0.731

*Significant at the 0.05 level (two-tailed).

**FIGURE 7:**
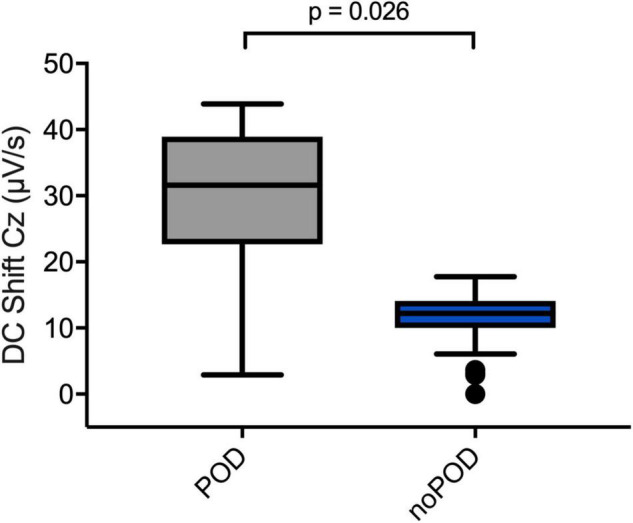
Direct current shifts in POD vs. noPOD patients. Boxplots comparing DC shifts at the Cz electrode at LOC in patients with and without postoperative delirium (POD vs. noPOD). Patients with postoperative delirium (POD) (*n* = 6) presented with significantly larger DC shifts compared to patients without delirium (noPOD) (*n* = 6). Non-parametric Mann–Whitney *U* test was used for group comparisons.

When comparing patients with and without postoperative delirium (POD vs. noPOD) significant differences of the DC shift at LOC could be observed. Of the patients whose perioperative DC-EEGs were examined, 8 (53.3%) developed POD. POD patients showed a significantly greater DC shift at LOC compared to patients without POD [POD 31.6 (22.7; 38.9) μV/s vs. noPOD 4.7 (2.2; 12.5) μV/s, *P* = 0.026] (Figure 7).

To adjust for possible confounders influencing the DC shift and POD (such as age, pre-existing comorbidities, and dosage of propofol for IOA) we conducted logistic regression models (Table 3).

**TABLE 3:** Logistic regression models.

Predictors	OR (95% CI)	*P*-value
	**Nagelkerke’s *R*^2^: 0.683**	
DC shift at LOC	1.81 (1.00; 1.39)	0.05
	**Nagelkerke’s *R*^2^: 0.770**	
DC shift at LOC	1.22 (0.99; 1.52)	0.063
Preoperative CCI	0.57 (0.16; 2.07)	0.392
	**Nagelkerke’s *R*^2^: 0.830**	
DC shift at LOC	2.26 (0.43; 11.93)	0.337
Age (years)	6.07 (0.98; 375.97)	0.392
	**Nagelkerke’s *R*^2^: 0.721**	
DC shift at LOC	1.91 (0.98; 1.46)	0.078
Propofol dosage at induction (mg)	1.03 (0.95; 1.12)	0.471

### Blood Analyses

The laboratory analyses revealed a correlation between the amplitude of the DC shift at LOC and CRP concentration on the day of the surgery. The larger the DC shift was, the higher the CRP concentration (Table 4). No significant correlation could be observed between the DC shift at LOC and markers related to activation or endothelial damage of the BBB (Table 4).

**TABLE 4:** Correlation of DC shifts at LOC (μV) and laboratory parameters.

	Spearman’s rho	*P*-value
Leukocytes (ng/nL)	0.478	0.098
CRP (mg/L)	0.577*	0.039*
IL-6 (pg/mL)	0.583	0.099
S100 (ng/mL)	0.269	0.374
Endocan (ng/mL)	0.286	0.493
Zonulin (ng/mL)	0.511	0.074
Leptin (ng/mL)	0.168	0.602

*Significant at the 0.05 level (two-tailed).

## Discussion

We found that DC shifts can be observed during HV and IOA in routine clinical practice. DC shifts during IOA were significantly greater at the time of LOC than before or thereafter. POD patients showed significantly greater DC shifts than noPOD patients during IOA.

In our study, HV and the subsequent decrease of pCO_2_ caused a DC shift in awake patients during routine clinical practice. This is in line with data from previous studies, which found similar shifts in both animals and humans and concluded that these shifts are of a non-neuronal origin generated by an altered electrical polarization across the BBB (Voipio et al., 2003; Kang et al., 2013). For example, Lehmenkuhler et al. (1999) found in scalp recordings in rats that hypercapnia induced large negative DC shifts, whereas hypoxia resulted in positive DC shifts with a negative deflection during recovery. Recordings at the surface of the cerebral cortex each showed opposite polarities to those at the scalp. It is assumed that the proton gradient across the BBB plays the critical role for these hypercapnia- and hypoxia-induced DC potentials in rats, rabbits, goats, and dogs (Kang et al., 2013). In cats and monkeys, DC shifts to hypercapnia at the scalp were inverted compared with scalp DC shifts in rats (Woody et al., 1970). Although these authors assumed that the DC shifts also arise at the BBB in cats and monkeys, they considered the proton gradient at the barrier to be less important for the potential generation than in the other species and rather suspected that the DC potential changes in cats and monkeys have a relationship to the hypercapnia-induced CBF changes that arise as a consequence of acidosis (Besson et al., 1970; Woody et al., 1970; Nita et al., 2004).

An intact BBB maintains a transendothelial potential difference between blood and brain tissue (Held et al., 1964). During a steady baseline state, the blood is negative to brain tissue by 1–5 mV, and changes to this voltage gradient, caused for example by pCO_2_ changes result in DC-EEG shifts (Sorensen et al., 1978; Voipio et al., 2003). Such DC shifts cannot only be observed in controlled conditions, but we found that they can also be recorded in regular patients during routine clinical care.

From the homogeneous DC shifts that arise at the BBB, e.g., in response to hypercapnia, it is necessary to distinguish strongly zonal DC shifts that arise from ion shifts across cell membranes. Most important here are the large intracortical DC shifts in the context of SDs and the significantly smaller DC shifts in the context of epileptic seizures (Dreier, 2011). During SD, regional depolarizations in individual neurons establish longitudinal gradients of depolarization and transmembrane current loops that sum in the extracellular space to build the negative DC signal (Dreier et al., 2013). This is accompanied by an almost complete breakdown of transmembrane ion gradients and neuronal cytotoxic edema (Lemale et al., 2022). Extracellular DC shifts during epileptic seizures, like SD, are also characterized by a highly zonal laminar profile, but they are associated with much smaller ion shifts than SD (Lemale et al., 2022). In addition, current source density analyses showed clear differences in the laminar profiles of DC shifts between SDs and seizures which underlines that these are two very different classes of phenomena (Wadman et al., 1992; Makarova et al., 2010). Relatively, glial cells appear to make a greater contribution to potential genesis in seizures (Dietzel et al., 1989). The essential commonality of the negative DC shifts in SDs and electrographic seizures is that in both cases there is a loss of cations across the cell membranes of neurons and glial cells into the intracellular compartment and precisely no relevant ionic shift across the BBB. Accordingly, if DC shifts of SDs and seizures are measured in brain slices lacking an intact BBB, they are virtually unchanged from their appearance in the intact animal or patient, in contrast to DC shifts to hypercapnia (Dreier and Heinemann, 1991; Maslarova et al., 2011; Kang et al., 2013). Ultimately, an extracellular electrode against a reference electrode outside the central nervous system (CNS) during SD or seizure measures this loss of cations from the extracellular to the intracellular space as a negative DC shift.

The DC signal during hypoxia is comparatively more complex. Hypoxia induces tissue acidosis early on (Mutch and Hansen, 1984; Taylor et al., 1996; Oliveira-Ferreira et al., 2010). In contrast to hypercapnia, in which intravascular pH drops more than brain interstitial pH (Kang et al., 2013), hypoxia is thought to drop interstitial pH more than intravascular pH, which would explain well why the DC shifts to hypercapnia and hypoxia are mirror images in the rat (Lehmenkuhler et al., 1999). However, in addition to this, hypoxia induces SDs in the further course (Dreier et al., 2019). Even though the DC shifts of SDs in humans are more than 30 times smaller at the scalp than in the subdural space this could add to the complexity of the signals (Drenckhahn et al., 2012; Hund et al., 2022). Thus, DC shifts to hypoxia have generators at different interfaces, i.e., they arise at both BBB and cell membranes. Important to note is overall that both the large DC shifts that occur in the brain during SDs and the smaller DC shifts that occur during seizures are difficult to measure at the scalp, whereas the DC potentials that arise at the BBB can be measured very well there.

In the present study, we demonstrated for the first time that DC shifts occur during anesthetic induction and that they are most pronounced around the time of LOC. An explanation for the occurrence of DC shifts during IOA, especially at LOC, could be related to circulatory and respiratory changes during this time. Typically, patients lose not only consciousness but also their respiratory drive during IOA. The resulting brief hypoventilation with hypercapnia until the anesthesiologist begins mask ventilation or intubation and mechanical ventilation could be the cause of the observed DC shifts, thus, following similar mechanisms as observed during the preoperative HV.

Besson et al. (1970) reported a strong dependence of DC shifts in cats and monkeys caused by changes to the CBF rather than the proton gradient across the BBB. They found that artificial increases in CBF were associated with negative DC shifts and decreases in CBF with positive DC shifts. These findings are in line with data published by Vanhatalo et al. (2003), who found an association between hemodynamic changes in brains of healthy human subjects and DC shifts. Many patients present with (brief) periods of hypotension in response to administration of anesthetic drugs. Thus, a possible explanation for the stronger DC shifts observed in this study at the time of LOC in POD patients could be a reduction in CBF due, for example, to impaired cerebral autoregulation making the brain more vulnerable to (brief) phases of hypotension. The resulting decreased supply of oxygen and essential nutrients to the brain could be a trigger for the occurrence of POD in these patients. While results of many previous studies point toward an association between intraoperative hypotension and POD, so far, evidence from these studies is not completely consistent and the underlying pathophysiological relationships are not sufficiently understood. Hirsch et al. (2015) were able to show that increased blood pressure fluctuation, not absolute or relative hypotension, was predictive of POD in elderly patients after non-cardiac surgery. A recent study by Wachtendorf et al. (2022) found that intraoperative hypotension within a range frequently observed in clinical practice (MAP <55 mmHg) is associated with increased odds of delirium after surgery.

We were unable to rule out or confirm direct associations between the observed DC shifts and hypotension and subsequent alterations to CBF or hypercapnia during IOA in our study as the patients included received an arterial catheter to monitor their blood pressure continuously only after IOA and intubation. During the induction period blood pressure was monitored *via* non-invasive measurements with 2 min intervals. We determined the arterial paCO_2_ immediately after establishing the arterial catheter, this, however, was several minutes after the examined EEG segments. Future studies should consider the placement of an arterial catheter prior to IOA for continuous blood pressure measurement during induction, as well as repetitive close-meshed blood gas analyses during this time.

The POD is one of the most common complications after surgery especially in older patients and is associated with higher rates of morbidity and mortality, however, the underlying pathophysiological mechanisms causing POD have not been completely understood (Ely et al., 2001, 2004; Pisani et al., 2009). Several studies suggest that neuroinflammation as well as injury to the BBB might contribute to the pathogenesis of POD. The BBB tightly regulates the movement of ions, molecules, and cells between the blood and the CNS. This barrier is crucial to provide the appropriate environment for proper neural function, and to protect the CNS from injury and disease. Disruption of the BBB under pathological conditions can for example enable the extravasation of immune cells and pro-inflammatory cytokines into the brain. In inflammatory states, which can be triggered by surgery for example, the endothelial activation leads to microcirculatory blood flow abnormalities and leukocyte adhesion on the BBB. This may contribute to increased BBB permeability and finally to neural tissue injury (Hughes et al., 2016). Hughes et al. (2016) were able to show that higher plasma markers of endothelial activation and BBB injury were associated with increased duration of delirium in critically ill patients. There are several other studies demonstrating an association between elevated levels of plasma markers of BBB disruption or pro-inflammatory cytokines and delirium during critical illness (Terrando et al., 2010; Tomasi et al., 2017; Saxena and Maze, 2018). Surgery activates the immune system causing an activation of immunocompetent cells and a release of proinflammatory mediators. These processes are thought to negatively affect the BBB, resulting in endothelial dysfunction and infiltration of peripheral cells and cytokines into the brain parenchyma (Subramaniyan and Terrando, 2019). In animal models, surgery was shown to upregulate enzymes that break down extracellular matrix, and lead to BBB opening and neuroinflammation. We were able to show a correlation between the amplitude of the DC shift observed at LOC and the CRP concentration observed on the day of the surgery. Since DC shifts could be associated with a dysfunction of the BBB, this finding supports the hypothesis of inflammation affecting the integrity of the BBB and the risk to develop a POD (Knaak et al., 2019). However, we were not able to find a correlation between DC shifts and markers of BBB endothelial activation in our study, which could be related to the small number of patients included in our study.

The pathophysiology of POD is multifaceted, complex, and not yet fully understood. Predisposing risk factors, i.e., higher age, polypharmacy, or pre-existing cognitive deficits, may contribute to increased brain vulnerability in some patients (Aldecoa et al., 2017). During surgery, other noxious stimuli such as inflammation, anemia, decreased perfusion, and decreased insulin sensitivity further increase brain vulnerability. The greater DC shifts observed in this trial in patients with POD contribute to further elucidate the pathophysiology of POD, as they hint toward an increased susceptibility to changes in gas tensions or CBF in these patients or indicate a dysfunction of the BBB. Hence greater DC shifts seem to be an EEG marker of an increase brain vulnerability in patients at higher risk to develop POD. However, further studies with larger sample sizes are needed to support this thesis.

Unlike a previous study conducted by Voipio et al. (2003), patients in our study did not show uniform polarity of their HV-induced DC shifts. This is consistent, however, with data from Vanhatalo et al. (2003) who investigated DC shifts in response to altered CBF in human subjects. They, too, found DC shifts with inter-individual differences in polarity and attributed this to differences in the time course, anatomical distribution, and the relative contribution of regional variations of intracranial pressure and changes to CBF (Vanhatalo et al., 2003). Another explanation for the different polarities in our study could be the position of the reference electrode, since it was not positioned above the mastoid but at the Fpz position.

There are some limitations to this study. First, the small sample size of 15 patients, who are all aged 65 or older limits the generalizability of our results. Even though we found no significant difference in regard to known POD risk-factors (as age, comorbidities, preexisting cognitive abilities), based on our small sample size we were not able to adjust our results to all these possible confounding factors. As the DC-EEG-measurements were performed during routine clinical care, Ag/AgCl skin electrodes were used and even though electrode impedance were kept below 5 kΩ., the skin’s galvanic response might have caused inaccuracies of the results.

## Conclusion

Direct current shifts can be observed during HV and IOA in routine clinical practice. During IOA, the shift was greatest at the time of LOC. Notably, POD patients showed significantly greater DC shifts at the time of LOC, presumably related to more pronounced hypotension, stronger alterations in gas tensions, BBB dysfunction, or a combination of these factors.

## Data Availability Statement

The raw data supporting the conclusions of this article will be made available by the authors, without undue reservation.

## Ethics Statement

The studies involving human participants were reviewed and approved by the Ethikkommission der Charité – Universitätsmedizin Berlin, Berlin, Germany. The patients/participants provided their written informed consent to participate in this study.

## Author Contributions

CS, SK, and VW conceived and designed the experiments. VW, SK, and GL performed the experiments. VW, JD, SM, GL, and SK analyzed the data. VW and SK wrote the first draft of the manuscript. All authors contributed to manuscript revision, read, and approved the submitted version.

## Conflict of Interest

CS was an inventor on patents, she reports grants during the conduct of a study from European Commission, from Aridis Pharmaceuticals Inc., B. Braun Melsungen, Drägerwerk AG & Co. KGaA, German Research Society, German Aerospace Center, Einstein Foundation Berlin, European Society of Anesthesiology, Federal Joint Committee, and Inner University grants. Grants promoting Science and Education from WHOCC, Baxter Deutschland GmbH, CytoSorbents Europe GmbH, Edwards Lifesciences Germany GmbH, Fresenius Medical Care, Grünenthal GmbH, Masimo Europe Ltd., and Pfizer Pharma PFE GmbH. Personal fees from Georg Thieme Verlag, Dr. F. Köhler Chemie GmbH, Sintetica GmbH, European Commission, Stifterverband für die deutsche Wissenschaft e.V./Philips, Stiftung Charite, AGUETTANT Deutschland GmbH, AbbVie Deutschland GmbH & Co. KG, Amomed Pharma GmbH, Touch Health, Copra System GmbH, Correvio GmbH, Max-Planck-Gesellschaft zur Förderung der Wissenschaften e.V., Deutsche Gesellschaft für Anästhesiologie & Intensivmedizin (DGAI), Medtronic, Philips Electronics Nederland BV, BMG, and BMBF. SK was an inventor on patents. Received speaker’s honoraria from Medtronic. She reports a grant during the conduct of a study from the German Research Society. The remaining authors declare that the research was conducted in the absence of any commercial or financial relationships that could be construed as a potential conflict of interest.

## Publisher’s Note

All claims expressed in this article are solely those of the authors and do not necessarily represent those of their affiliated organizations, or those of the publisher, the editors and the reviewers. Any product that may be evaluated in this article, or claim that may be made by its manufacturer, is not guaranteed or endorsed by the publisher.

## Supplementary Material

Supplementary Figure 1 Electroencephalography-electrode placement. Electrodes were placed on the patients’ head in accordance with the 10/20 system at Fp1, Fp2, T3, T4, Cz, O1, and O2 with reference electrode at Fpz.
