# Supplementary material for: Increased Direct Current-Electroencephalography Shifts During Induction of Anesthesia in Elderly Patients Developing Postoperative Delirium
Source: Front Aging Neurosci. 2022 Jun 28;14:921139. doi: 10.3389/fnagi.2022.921139 (PMC9274126; doi:10.3389/fnagi.2022.921139)
Supplement: Supplementary file 1 [file Table_1.DOCX]

| Patient No. | Surgery |
| --- | --- |
| 1 | Debulking surgery for ovarian cancer |
| 2 | Extended left hemihepatectomy |
| 3 | Adrenalectomy |
| 4 | Pylorus preserving pancreaticoduodenectomy |
| 5 | Posterior laminectomy and stabilization of the spine |
| 6 | Pylorus preserving pancreaticoduodenectomy |
| 7 | Pylorus preserving pancreaticoduodenectomy |
| 8 | Tumor resection and implantation of an inverse humeral prosthesis |
| 9 | Atypical liver resection |
| 10 | Trisectorectomy (extended liver resection) |
| 11 | Femoral thromboendarterectomy |
| 12 | Femoral thromboendarterectomy |
| 13 | Laparoscopic pancreatic tail resection and splenectomy |
| 14 | Debulking surgery for ovarian cancer |
| 15 | Debulking surgery for ovarian cancer |

Supplement Table 1. Performed surgeries
