# Supplementary figures and images for: Increased Direct Current-Electroencephalography Shifts During Induction of Anesthesia in Elderly Patients Developing Postoperative Delirium
Source: Front Aging Neurosci. 2022 Jun 28;14:921139. doi: 10.3389/fnagi.2022.921139 (PMC9274126; doi:10.3389/fnagi.2022.921139)

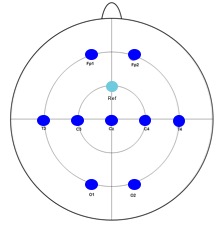

Supplement: Supplementary Figure 1 — Electroencephalography-electrode placement. Electrodes were placed on the patients’ head in accordance with the 10/20 system at Fp1, Fp2, T3, T4, Cz, O1, and O2 with reference electrode at Fpz. [file Image_1.JPEG]
